# Occurrence of Lupus-Like Nephritis in a Girl With NLRC4 Gain-of-Function Mutations

**DOI:** 10.1016/j.ekir.2024.01.061

**Published:** 2024-02-02

**Authors:** Franck Zekre, Audrey Laurent, Bruno Ranchin, Anne-Perrine Foray, Marine Thilloy, Noémie Laverdure, Cécile Picard, Alexandre Belot

**Affiliations:** 1 Pediatric Rheumatology - Nephrology and Dermatology Unit, Hôpital Femme Mère Enfant, Hospices, Civils de Lyon, Bron, France; 2 Immunology Department, Lyon Sud University Hospital, Pierre Bénite, France; 3 Service de gastroentérologie, hépatologie et nutrition pédiatriques, Hôpital Femme Mère Enfant, Hospices Civils de Lyon, Bron, France; 4 Institut de pathologie Multisite, Groupement hospitalier Est, Hospices Civils de Lyon, Bron, France; 5 International Center of Infectiology Research (CIRI), INSERM U1111, CNRS, UMR5308, ENS of Lyon, Claude Bernard University Lyon 1, Lyon, France

**Keywords:** glomerulonephritis, interleukin-18, lupus nephritis, mutation, NLRC4 GOF

## Introduction

NLRC4 belongs to the family of innate immunity receptors. These receptors have the ability to detect microbial patterns and molecular signals of cellular damage within the cell. The involvement of NLRC4 in autoinflammatory pathologies was highlighted in 2014, when infantile enterocolitis and macrophagic activation syndrome were reported in patients with gain-of-function mutations in NLRC4.^1^ Therefore, biologically, the disease is characterized by increased production of interleukin (IL)-1β and IL-18 (Table 1), justifying the use of anti-IL-1 biotherapy in the management of NLRC4 mutations. A recent report identified a pathogenic variant in NLRC4 associated with clinical features of systemic lupus erythematosus (SLE) in 1 patient, presenting with recurrent fever, enteritidis, macrophages activation syndrome, and lymphoproliferation biological features of SLE.^2^

**Table 1:** Teaching point

NLRC4-GOF	NLRC4 serves as a constitutive component of the inflammasome, a protein complex crucial to innate immunity. The primary function of the inflammasome is the maturation of the proinflammatory cytokines, interleukin 1 beta (IL-1 β) and interleukin 18 (IL-18), through proteolytic cleavage. This process is pivotal for triggering a hyperinflammatory state essential for the elimination of pathogens. GOF mutations in NLRC4 have been identified as an autoinflammatory disease, leading to an increased production of IL-18 and IL-1 β. This rare inflammatory disease may promote full-house nephropathy.
Nonlupus full house nephropathy	This is an anatomopathological diagnosis characterized by the presence at glomerular level of lesions observed in systemic lupus disease (complement and Ig deposits), with no antibodies found in the blood. To date, the origin of these lesions remains unknown, and the pathophysiological mechanisms are not entirely clear.

GOF, gain-of-function.

To date, no renal complications have been reported in association with NLRC4 gain-of-function mutations. We present the case of a young patient who presented with a full house glomerulonephritis evocative for a SLE. This unexpected renal involvement raises interesting questions about the potential role of NLRC4 and elevated IL-18 in systemic autoimmunity.

## Case Presentation

A 3-year-old girl of Caucasian origin, was diagnosed with an autoinflammatory disease at the age of 6 months by *NLRC4* mutation at exon 4, variant c.620G>A (p. Arg207Lys). She presented with persistent fever, macrophagic activation syndrome, diffuse urticarial rash, and inflammatory enteropathy. The therapeutic management of her autoinflammatory disease consisted of the initiation of a corticosteroid therapy initially at 2 mg/kg/day and then gradually tapered, with the addition at the age of 6 months of a biologic therapy neutralizing the activity of IL-1, anakinra, in subcutaneous injection at 4 mg/kg/d, and after canakinumab, with a subcutaneous injection every 4 weeks at a dose of 7 mg/kg. This strategy resulted in a complete remission of the disease.

**Figure 1:**
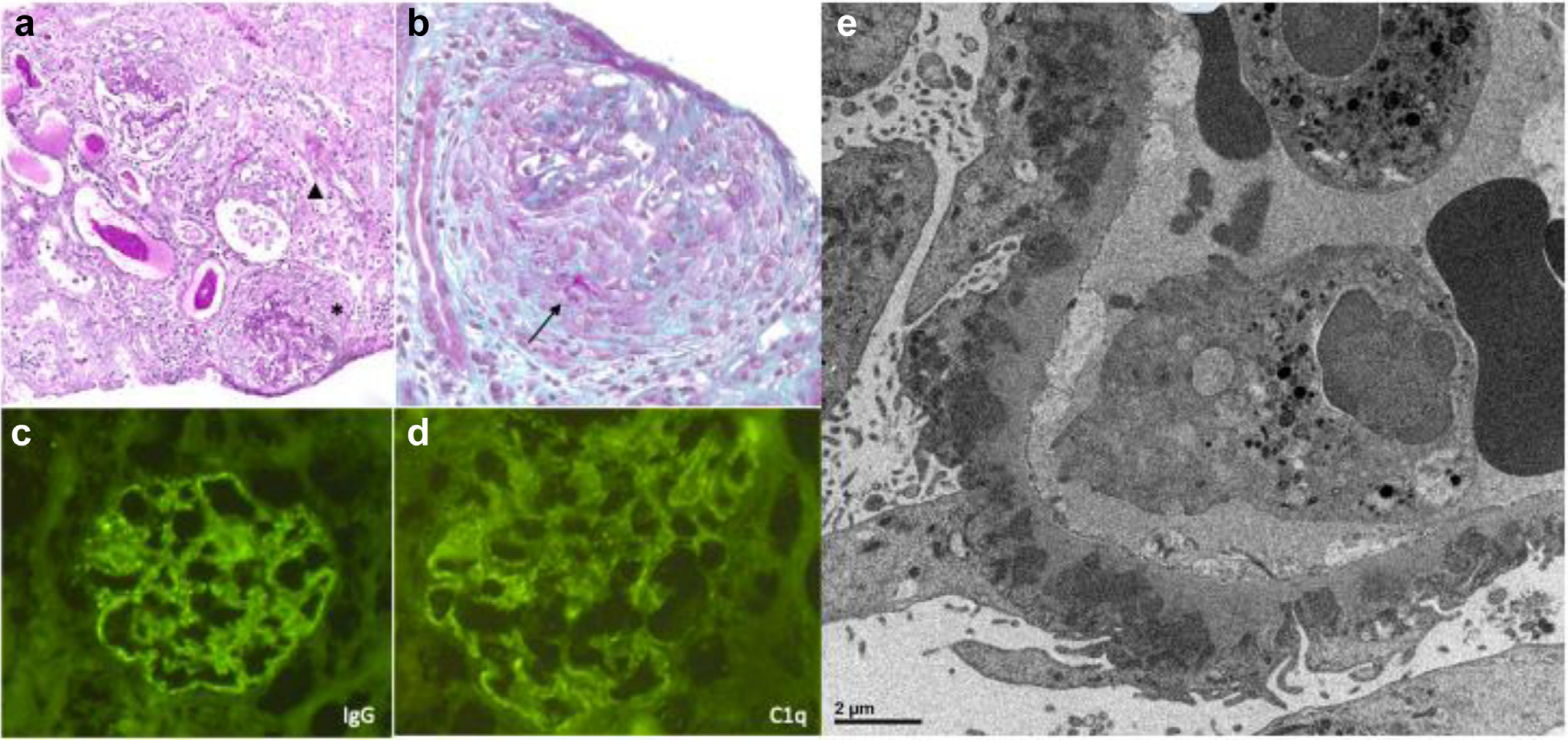
Kidney biopsy of the patient. (a and b: optical microscopy) (a) Periodic Acid Shift: staining showing association of active and chronic glomerular lesions such as circumferential crescent (∗) and segmental fibrosis (Δ). Interstitium displays edematous fibrosis and mild inflammation containing plasma cells. (b) Circumferential crescent with segmental fibrinoid necrosis on trichrome stain (→). (c) Detection of IgG membranous coarse deposits by immunofluorescence. (d) Capillary wall deposits stain for C1q in the same membranous pattern, highly suggestive of a superimposed class V lupus nephritis. (e) Electron microscopy showing amorphous subepithelial electron-dense deposits. There was extensive foot process effacement with focal microvillous transformation. No tubuloreticular inclusions were found in endothelial cells.

At the age of 3 years, the girl presented with hematuria, oliguria, abdominal pain, and edema in the lower limbs. The blood test performed during this episode showed an anemia with hemoglobin at 78 g/l, platelets of 332 G/l, white blood cells of 7.85 G/l, C-reactive protein of 8 mg/l, sedimentation rate of 76 at the first hour, albumin of 11 g/l, and creatinine of 30 μmol/l. Urinalysis showed nephrotic proteinuria of 11.21 g/l and a urinary protein-to-creatinine ratio of 20 g/g. The complement C3 and C4 levels were normal and the immunological analysis did not reveal any antinuclear antibodies or other antibodies involved in connectivitis. Furthermore, the transcriptomic signature of interferon was negative, whereas the IL-18 dosage was 37,500 pg/ml (standard between 45 and 330 pg/ml). Kidney biopsy was performed. The histological examination (Figure 1) revealed, a diffuse proliferative glomerulonephritis, including active glomerular lesions such as cellular crescents involving nearly half of the glomeruli, focally associated with fibrinoid necrosis, and associated with neutrophils-rich endocapillary hypercellularity. Granular subepithelial deposits with silver-positive “spikes” were also observed, revealing a superimposed membranous glomerulonephritis. A few chronic glomerular lesions with focal segmental sclerosis and fibrous crescents were also noticed. There was a mild interstitial fibrosis and tubular atrophy, and a significant interstitial inflammation was showed, composed of T-cells and plasma cells. The vessels were unremarkable. Immunofluorescence analysis revealed a “full house” pattern nephritis with membranous deposits of IgG (IgG1 and IgG3 predominant), IgM, Kappa and Lambda light chains, as well as abundant fractions of the C3 component, and to a lesser extent of the C1q complements components. Very few IgG deposits were noted in the sub-endothelial area.

Electron microscopy revealed amorphous subepithelial electro-dense deposits, as well as diffuse podocytes foot-process effacement. There were no obvious deposits within the tubular or vascular compartments, and no tubuloreticular inclusions (the so-called interferon footprint), were found. Taken together, histological findings of the patient highly suggested a diffuse proliferative lupus nephritis with superimposed membranous glomerulonephritis (ISN/RPS class IV + V). Exostosin 2, epidermal neural growth factor-like 1, THSD7A, and PLA2R1 antigen immunostaining were also negative. A Congo red staining was performed, showing no amyloid deposition.

In view of this renal impairment, the patient received combining boluses of corticosteroids at 1 g/1.73 m^2^ over 3 days followed by a relay of oral corticosteroids after the boluses. In addition, treatment with mycophenolate mofetil at 1200 mg/m^2^/d and with hydroxychloroquine at 6 mg/kg/d was initiated. Canakinumab was continued alongside the conventional treatment. After 3 months of treatment, IL-18 values stabilized at ∼10,000 pg/ml, proteinuria normalized and the protein-to-creatinine ratio in urine decreased to 0.36 g/g.

## Discussion

In a total of 30 reported NLRC4 gain-of-function patients, no kidney lesions were identified. Full house pattern is consistent with lupus nephritis, but a number of other conditions have been associated with this aspect by immunofluorescence representing “non-lupus full house nephropathy” entity (Table 1). Rijnink *et al.*^3^ did not observe the occurrence of systemic lupus in any of the 32 patients of the cohort with non-lupus full house nephropathy after a median follow-up of 20 years.

In the context of our patient, different hypotheses were explored regarding the underlying cause of renal damage. One hypothesis was a lupus-like glomerulopathy induced by the biologic therapy, canakinumab. Drug induced SLE have been reported, often characterized by the presence of anti-nuclear antibodies, specifically anti-histone antibodies. However, the extensive use of canakinumab in autoinflammatory diseases has never been associated with this glomerulopathy. Moreover, in our patient, no antibodies typically associated with drug induced lupus were detected.

Another hypothesis consisted of a direct role of NLRC4 defects in the development of kidney damage. Indeed, although the pathophysiological mechanisms are yet to be clarified, it is known that the NLRC4 inflammasome is directly involved in the renal tubular epithelial lesions responsible for diabetic nephropathy. The level of NLRC4 expression is directly correlated to renal damage in diabetic nephropathy.^4^ Furthermore, the correlation between IL-18 levels and renal involvement was discussed as a potential explanation for the observed renal damage. IL-18 is a proinflammatory cytokine involved in various pathophysiological processes related to renal damage. Similarly, a correlation between inflammatory infiltrate in renal biopsies, elevated proteinuria, and increased serum IL-18 levels has been shown in IgA nephropathy.^5^

Finally, another hypothesis strengthen by the recent observation of Wan *et al.*^2^ was a potential link between the NLRC4 mutation and lupus development. Patients with active renal involvement in SLE have higher serum IL-18 levels than those without renal involvement. Some studies mention that IL-18 may serve as a useful assay for assessing the severity of renal involvement in SLE. Animal models of autoimmune diseases have demonstrated that suppression of IL-18-mediated signaling can protect against renal damage, particularly glomerulopathy. In this context, the elevation of IL-18 in our patient might have contributed to the glomerular damage.^6^ Interestingly, the interferon signature remains negative in our patient, suggesting that interferon signaling is not necessary for lupus development in this context.

In light of a recent discovery of a young adult with NLRC4 gain-of-function mutation who exhibited autoimmune features consistent with lupus,^2^ this additional finding suggest a potential role of IL-18 in the development of certain forms of lupus nephritis and autoimmunity. This information is particularly noteworthy because it introduces a novel druggable pathway previously unrecognized in the management of SLE.

## Disclosure

All the authors declared no competing interests.

## Patient Consent

Informed consent was obtained and an information sheet on the use of the data was delivered to the patient and family.
